# The Queen and the Dark Twin: Heme, Protoporphyrin IX, and State Transitions in Liver Metabolism

**DOI:** 10.3390/molecules31101719

**Published:** 2026-05-19

**Authors:** Swamy R. Adapa, Rays H. Y. Jiang

**Affiliations:** 1 Department of Global, Environmental, and Genomic Health Sciences, College of Public Health, University of South Florida, Tampa, FL 33612, USA; 2 Genomics Program, University of South Florida, Tampa, FL 33612, USA

**Keywords:** heme, porphyrin, protoporphyrin IX (PPIX), metabolic flux, liver metabolism, hepatocellular carcinoma, ferrochelatase (FECH), aminolevulinate synthase (ALAS1), cytochrome P450

## Abstract

Heme metabolism in the liver has traditionally been described as a linear pathway that supports oxygen utilization, redox balance, and detoxification. Here, we synthesize recent evidence and propose a framework in which heme functions as a system-level regulator, the “queen” of metabolism, whereas its upstream intermediate protoporphyrin IX (PPIX) represents a chemically reactive “dark twin” that emerges when metabolic flux fails to resolve. In this view, metabolic state is defined not only by end products but also by the behavior of pathway intermediates. This system is spatially organized. Hepatocytes dominate heme synthesis and utilization. In contrast, liver stromal compartments, particularly Kupffer cells, play a central role in heme degradation through heme oxygenase-1 (HMOX1), linking heme turnover to iron recycling and stress adaptation. The metabolic state of the liver therefore reflects not only pathway flux but also the degree of coupling between these cellular compartments. We propose a state model of hepatic heme metabolism. In the resolution state, most evident during inflammation, coordinated hepatocyte–macrophage activity maintains flux and limits intermediate accumulation. In contrast, the expansion state, exemplified in cancer, is defined by impaired flux completion, leading to PPIX accumulation, metabolic heterogeneity, and oxidative stress. This framework reframes liver disease through intermediate behavior rather than pathway presence: porphyrias reflect direct overload, metabolic liver diseases partial expansion, and hepatocellular carcinoma a fully developed expansion state. By focusing on the “intermediate space,” this model links biochemistry, spatial organization, and disease pathogenesis, while suggesting new opportunities for diagnosis and therapy based on metabolic state.

## 1. Introduction: The Queen and the Dark Twin

Metabolism is often framed in terms of a limited set of molecular “currencies,” including ATP [1], NAD^+^/NADH [2], and acetyl-CoA [3], which function as broadly utilized carriers of energy, electrons and carbon units across metabolic reactions. While these molecules enable metabolic transactions, they do not directly determine how metabolic systems are organized, prioritized, or coordinated across cellular functions. In contrast, heme occupies a distinct role. As an iron-coordinating cofactor embedded within proteins that govern respiration [4], detoxification [5], and redox control [6,7], heme integrates multiple layers of metabolism into a coherent functional system [6,7,8]. We therefore propose that heme is not simply another metabolite, but may function as a system-level regulator, the “queen” of metabolism. We use the term ‘queen’ not to imply exclusivity among metabolic regulators, but to emphasize heme’s unique role as a flux-resolved cofactor that directly couples pathway completion to functional output, particularly in hepatocytes.

The distinction is not merely semantic. ATP and NAD^+^ function as broadly interchangeable carriers whose abundance reflects cellular energetic and redox states [3,9,10] but does not, in itself, encode completion of specific metabolic pathways. In contrast, heme represents the end product of a tightly regulated biosynthetic sequence, whose successful incorporation into hemoproteins signifies that metabolic flux has been resolved into functional output [11]. In hepatocytes, this integration is particularly pronounced: heme supports cytochrome P450 (CYP)–mediated detoxification, mitochondrial respiration, and antioxidant defense [5], positioning it at the intersection of environmental response and cellular homeostasis.

However, this system also reveals an alternative outcome when metabolic flux fails to resolve [12]. Immediately upstream of heme lies protoporphyrin IX (PPIX), a chemically reactive intermediate that accumulates when the terminal step of heme synthesis, catalyzed by ferrochelatase, is impaired. Extensive biochemical and clinical studies, particularly in the context of 5-aminolevulinic acid (5-ALA)–based photodynamic diagnosis and therapy, have demonstrated that PPIX preferentially accumulates in tumor tissues relative to adjacent normal regions, forming the basis for its use in fluorescence-guided imaging and treatment [13,14,15,16]. Unlike heme, which is efficiently incorporated into functional hemoprotein complexes, PPIX remains largely unbound, rendering it photoreactive, redox-active, and spatially heterogeneous [12,17]. We therefore refer to PPIX as the “dark twin” of heme: a product of the same biosynthetic pathway that reflects an alternative, incompletely resolved metabolic state.

This duality motivates a shift in perspective. Rather than defining metabolism solely by its end products, we propose that metabolic state may also be reflected in the behavior of pathway intermediates [18] and the efficiency of flux resolution. In the liver, this principle is further shaped by spatial organization: hepatocytes are the primary site of heme synthesis and utilization [5,12], whereas liver endothelial cells and macrophages, particularly Kupffer cells [19,20,21], mediate heme degradation and recycling. The balance, or disruption, of this intercellular coordination may therefore give rise to distinct metabolic states.

In this review, we synthesize recent studies on heme metabolism and the roles of its intermediates across physiological and pathological contexts, including metabolism, inflammation, and cancer [7,8,22,23,24,25,26]. Emerging evidence suggests that heme functions not only as a cofactor, but also as a signaling molecule involved in redox sensing and cellular regulation [27,28]. Drawing on biochemical, transcriptional, and spatial studies across liver physiology and disease, we propose a framework in which heme metabolism operates across different regimes: a resolution state, in which metabolic flux is completed or actively drained, and intermediates remain low, and an expansion state, in which flux is impaired, and intermediates such as PPIX accumulate. We further suggest that this transition can be understood across three levels: pathway dynamics, functional utilization, and spatial organization, linking metabolic flux to liver physiology and disease.

## 2. Pathway Logic: Flux Completion vs. Intermediate Accumulation

Heme biosynthesis is classically described as a linear, eight-step pathway spanning two cellular compartments. The first and final steps, catalyzed by aminolevulinate synthase 1 (ALAS1) [11,12,29] and ferrochelatase (FECH) [11,30], respectively, occur in mitochondria, while the intermediate reactions proceed in the cytosol. This architecture is not incidental: it enforces coordination between pathway entry, intermediate processing, and terminal completion. Under physiological conditions, this coordination is highly efficient [4,11,12,31]. Substrate input is matched to downstream capacity, intermediates remain transient, and flux is resolved into heme with minimal accumulation of upstream species.

**Figure 1:**
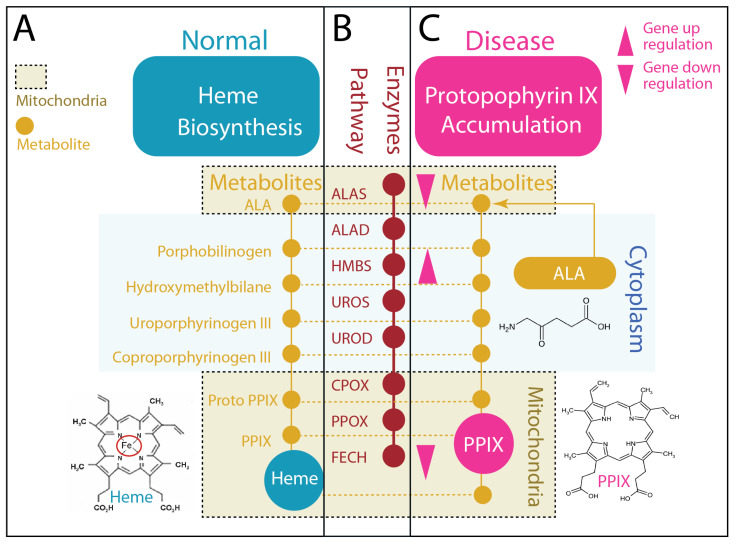
Heme biosynthesis in homeostasis versus PPIX accumulation states. (**A**): Normal heme biosynthesis (homeostatic state). Under physiological conditions, this pathway operates with high efficiency and tight coordination between substrate input and product formation. Metabolic flux is balanced, and pathway intermediates remain transient and low in abundance, allowing robust heme production without upstream accumulation. (**B**) Heme biosynthesis proceeds through a linear pathway of eight enzymatic steps distributed across two cellular compartments. The rate-limiting first enzyme, aminolevulinate synthase 1 (ALAS1), and the terminal enzyme, FECH, are localized in mitochondria, while the intermediate steps occur in the cytosol. (**C**) PPIX accumulation (pathological expansion state). Under pathological conditions, such as cancer, this coordinated flux is disrupted. Expression or activity of first and terminal steps, particularly ALAS1, is reduced, while upstream and intermediate enzymes (e.g., hydroxymethylbilane synthase, HMBS) may be maintained or upregulated, creating a functional bottleneck at the final step. As a result, heme production is inefficient, and upstream intermediates accumulate, most notably PPIX, which is minimal under normal conditions. This state reflects incomplete flux resolution and expansion of the intermediate pool. PPIX induction is achieved with exogenous aminolevulinic acid (ALA), bypassing the ALAS1-controlled first step. In the normal state, ALA supplementation increases heme output with minimal intermediate buildup. In contrast, in the pathological state, ALA exacerbates the bottleneck, leading to pronounced accumulation of PPIX.

This property, completion without accumulation, defines the homeostatic state (Figure 1A,B). Heme production in hepatocytes [5] can reach high levels to support CYP activity, mitochondrial respiration, and redox buffering, yet intermediate pools remain low. The pathway operates as a tightly coupled system [31] in which each step is constrained by the capacity of the next, preventing the buildup of reactive intermediates.

In contrast, pathological conditions reveal a distinct regime (Figure 1C). In hepatocellular carcinoma and related states, both longstanding biochemical studies [14,15] and recent single-cell and bulk transcriptomic analyses [23,24] indicate that coordination between pathway entry, intermediate processing, and terminal insertion is disrupted. Expression or activity of key regulatory enzymes, including ALAS1 and FECH, is often reduced, while intermediate enzymes such as hydroxymethylbilane synthase (HMBS) are maintained or upregulated [23,24]. This imbalance creates a functional bottleneck at the terminal step of heme synthesis. As a result, metabolic flux is no longer efficiently resolved into heme; instead, it accumulates upstream.

The consequence is the emergence of PPIX, which is minimal under normal conditions but becomes abundant when flux completion fails. Unlike heme, PPIX is not incorporated into stable protein complexes and remains chemically reactive, marking an incomplete metabolic state [17,25]. The pathway is not inactive; it is uncoupled.

Exogenous ALA provides a functional probe of this pathway logic. As ALAS1 catalyzes the rate-limiting first step of heme biosynthesis to produce ALA [11,29,32], exogenous ALA bypasses endogenous control of pathway entry and increases substrate input independently of cellular regulation. In the homeostatic state, this results in increased heme production with minimal accumulation of upstream intermediates. In contrast, under pathological conditions, ALA can exacerbate a terminal bottleneck, leading to pronounced accumulation of PPIX. Clinically, this property has been widely exploited, as ALA-induced PPIX accumulation enables fluorescence-guided imaging and photodynamic therapy across a range of cancers [13,14,15,16]. Thus, the response to ALA provides a functional readout of whether metabolic flux is efficiently completed or remains impaired.

Taken together, these observations redefine heme biosynthesis as a system governed not by pathway presence, but by flux resolution. Heme signifies completion. PPIX signifies failure of completion. The transition between these outcomes establishes the first axis of the “queen” and the “dark twin.”

## 3. Functional Consequences: Rewiring of Heme Utilization

**Figure 2:**
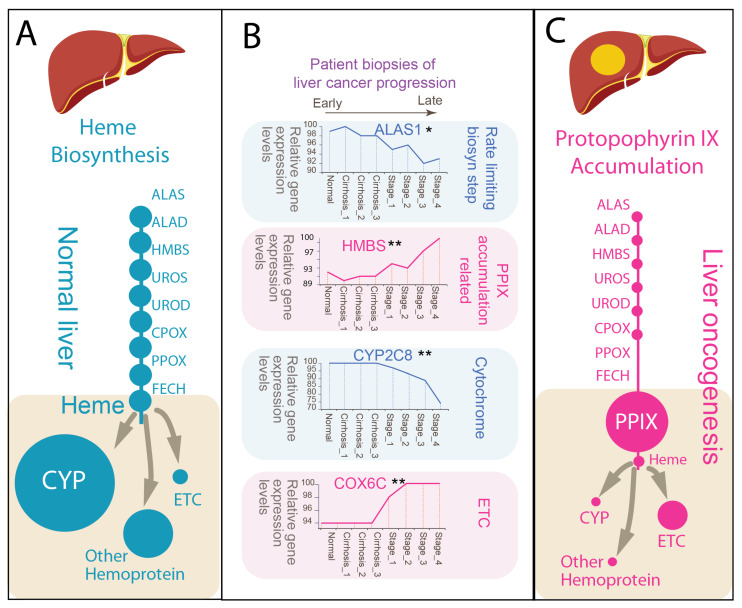
Heme homeostasis and PPIX accumulation define divergent metabolic states in normal and pathological liver. (**A**) Heme homeostasis in normal liver. Under physiological conditions, hepatic heme biosynthesis is balanced and efficient, supporting high-level production of heme as a functional cofactor. Heme is broadly incorporated into hemoproteins, including CYP, catalase (CAT), and components of the electron transport chain (ETC), rendering hepatocytes uniquely enriched in hemoproteins. This state reflects coordinated flux through the biosynthetic pathway, with minimal accumulation of upstream intermediates. (**B**) Transcriptional remodeling across disease progression. Gene expression analysis of human liver biopsies spanning normal liver, cirrhosis (stages 1–3), and hepatocellular carcinoma (HCC; cancer stages 1–4) reveals progressive disruption of heme biosynthetic balance. Data from Wurmbach et al. [33]. The rate-limiting enzyme ALAS1 is downregulated during oncogenic progression, while intermediate pathway enzymes such as HMBS are upregulated, indicating uncoupling between pathway entry and completion. Functional heme utilization is also reprogrammed: expression of major hepatic CYP genes (e.g., CYP2C8) declines in HCC, whereas mitochondrial ETC-associated genes (e.g., CYC1) are maintained or increased. These shifts suggest reduced capacity for canonical hepatic functions alongside preservation or enhancement of mitochondrial energy metabolism. (* *p* < 0.05; ** *p* < 0.01). (**C**) PPIX accumulation and altered heme utilization in cancer. In the pathological expansion state, impaired flux completion, particularly at the ferrochelatase step, leads to accumulation of the intermediate PPIX, which is minimal in normal liver. The reduced heme pool is differentially allocated, with diminished incorporation into CYP systems and relative prioritization toward mitochondrial ETC components. Thus, cancer cells exhibit both intermediate accumulation and selective heme utilization, reflecting a reprogrammed metabolic state.

If Figure 1 defines how metabolic flux is completed or stalled, the next question is how this difference propagates into cellular function. In the liver, heme is not merely an end product but a limiting cofactor that supports the capacity of major metabolic systems. Accordingly, changes in heme biosynthesis may be reflected not only in intermediate accumulation but also in the redistribution of functional output (Figure 2). Our work [23,24], together with prior studies [33], leveraging human liver transcriptomic datasets derived from surgical biopsies, provides insight into these changes across disease progression, from normal liver, through inflammation-dominant cirrhosis, to hepatocellular carcinoma [33].

In the homeostatic state, hepatocytes function as heme-rich cells. Transcriptomic analyses of human liver biopsies demonstrate robust expression of heme metabolic genes [23,24,34], consistent with the liver’s established role as a major site of endogenous heme production. Hepatic detoxification depends on a diverse array of CYP enzymes [5], while the liver’s high metabolic demand is supported by abundant mitochondrial activity [6]. Efficient completion of heme biosynthesis enables broad incorporation of heme into hemoproteins, including CYP enzymes, catalase, and components of the electron transport chain (ETC) (Figure 2A). This supports the liver’s capacity to sustain detoxification, oxidative metabolism, and redox buffering. Importantly, these functions can coexist because heme supply is sufficient and effectively distributed across cellular systems.

In addition to its incorporation into hemoproteins, a small fraction of intracellular heme exists as a labile or exchangeable pool [35]. This labile heme is not stably protein-bound and can participate in regulatory processes, including redox sensing and transcriptional control [27,36]. Although quantitatively minor relative to total cellular heme, this pool is functionally significant, acting as a signaling intermediate that links metabolic flux to cellular responses [37]. Under conditions of efficient flux completion, labile heme is tightly buffered and maintained at low levels. In contrast, when pathway coordination is disrupted, alterations in heme synthesis, utilization, or degradation may expand or destabilize this pool, amplifying redox signaling and contributing to metabolic stress [37].

During disease progression, this balance becomes progressively disrupted (Figure 2B). Gene expression analyses of human liver biopsy datasets, spanning normal tissue through cirrhosis to hepatocellular carcinoma (HCC), reveal a characteristic uncoupling between pathway entry, intermediate processing, and functional output. The rate-limiting enzyme ALAS1 is reduced, whereas intermediate enzymes such as HMBS are maintained or relatively increased, consistent with the bottleneck described in Section 2. At the functional level, this uncoupling is accompanied by divergence in heme utilization: expression of major CYP genes (e.g., CYP2C8) declines [38,39], whereas mitochondrial electron transport chain (ETC)–associated genes (e.g., COX6C) are preserved or increased [24,40]. Although the Warburg effect highlights increased glycolysis, mitochondrial respiration frequently remains active, reflecting metabolic reprogramming rather than complete suppression [40,41,42].

This shift reflects more than a simple reduction in total heme production. Rather, it suggests selective allocation of a constrained heme pool. As flux completion becomes inefficient, available heme may be preferentially directed toward essential processes such as mitochondrial respiration, while liver-specific functions, including xenobiotic metabolism, are diminished [38,39]. Concurrently, upstream intermediates, particularly PPIX, accumulate as a consequence of incomplete pathway resolution (Figure 2C).

The coexistence of reduced functional heme output, intermediate accumulation, and selective utilization is consistent with a reprogrammed metabolic state. In this context, cancer cells do not simply lack heme; rather, they operate under conditions in which heme is both limiting and redistributed [24], while upstream pathway activity continues to generate intermediates that cannot be fully resolved [43,44].

Accordingly, the distinction between the “queen” and the “dark twin” becomes functional. Heme supports integrated metabolic activity, whereas PPIX reflects a system in which this integration is impaired. The transition between these states is therefore expressed not only at the level of pathway flux, but also in the reorganization of cellular metabolism.

## 4. Spatial Organization: Hepatocyte–Macrophage Coupling

Heme metabolism in the liver is not uniformly distributed but is spatially organized across interacting cell types. Evidence from classical biochemical studies and more recent single-cell analyses highlights the cellular heterogeneity of the liver and the distinct metabolic roles of its major compartments [45,46,47]. In the human liver, hepatocytes constitute the dominant parenchymal population and serve as the primary site of heme synthesis and utilization. In contrast, non-parenchymal cells, particularly liver-resident macrophages (Kupffer cells) [46], represent a major functional compartment involved in immune surveillance and metabolic regulation, including heme degradation and iron recycling.

Although hepatocytes comprise the majority of liver cells, a smaller population of Kupffer cells plays a disproportionate role in heme degradation and iron recycling, supporting a functional division of labor between production and clearance [43,44]. While the spleen serves as the primary site of systemic erythrocyte turnover [48], the liver maintains an intrinsic heme recycling system in which Kupffer cells, along with endothelia, mediate heme degradation and iron recovery [21,44,48], complementing hepatocyte-driven heme synthesis.

This division of labor extends to heme metabolism: hepatocytes drive heme biosynthesis and incorporation into hemoproteins, whereas Kupffer cells mediate heme degradation and iron recycling. The metabolic state of the liver therefore emerges from the coupling between these compartments, linking heme production to clearance.

### 4.1. Homeostasis: Division of Labor

**Figure 3:**
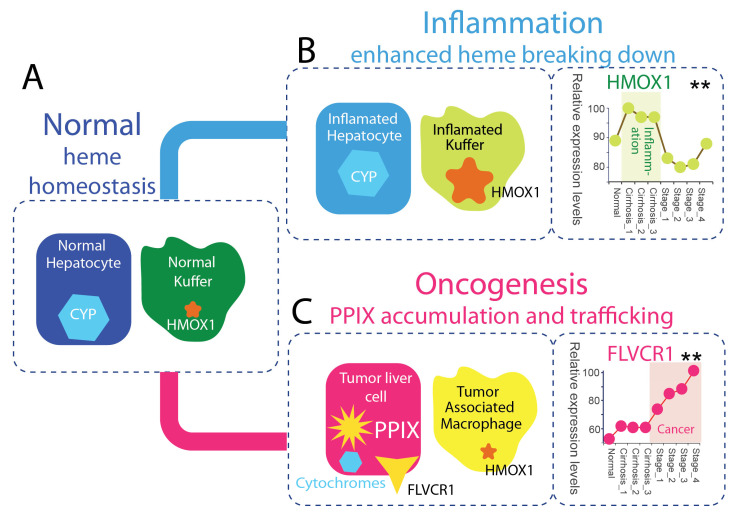
Spatial coordination between hepatocytes and macrophages defines heme metabolic states in the liver. (**A**) Homeostatic state. In a normal liver, heme metabolism is spatially partitioned between hepatocytes and macrophages. Hepatocytes are the dominant site of heme biosynthesis and utilization, expressing high levels of CYP enzymes and other hemoproteins. In parallel, resident liver macrophages (Kupffer cells) serve as the primary site of heme degradation, characterized by constitutive expression of HMOX1. This division of labor enables efficient heme turnover, with hepatocyte production balanced by macrophage-mediated clearance, maintaining low levels of upstream intermediates. ** shows *p* < 0.01) (**B**) Inflammatory (resolution) state. During inflammation, this spatial coordination is preserved but functionally shifted toward enhanced degradation. Hepatocytes maintain core metabolic functions, including CYP expression, while Kupffer cells exhibit marked upregulation of HMOX1, increasing heme breakdown and promoting iron recycling. Gene expression analysis across human liver biopsies, from normal to cirrhosis (stages 1–3), demonstrates induction of HMOX1 during inflammatory progression. This state is defined by effective coupling between hepatocyte flux and macrophage clearance, resulting in suppression of intermediate accumulation. (**C**) Oncogenic (expansion) state. In hepatocellular carcinoma (HCC), hepatocyte–macrophage coupling is disrupted. Transformed hepatocytes exhibit impaired heme biosynthesis, with reduced expression of CYP genes and accumulation of the upstream intermediate PPIX. Concurrently, genes involved in heme and porphyrin export, such as FLVCR1, are progressively upregulated across HCC stages (1–4), indicating increased trafficking of heme-related metabolites into the tumor microenvironment (*p* < 0.01). This state is characterized by intermediate accumulation, reduced functional heme utilization, and altered intercellular exchange. In parallel, resident macrophages are reprogrammed into tumor-associated macrophages (TAMs), which exhibit altered heme-handling and iron-regulatory functions, further weakening effective degradation and contributing to expansion of the intermediate pool. Together, these changes reflect a breakdown of coordinated heme homeostasis and the emergence of the expansion state.

In the healthy liver, heme homeostasis depends on a spatially organized division of labor (Figure 3A). Hepatocytes dominate heme biosynthesis and heme utilization, channeling heme into CYPs, catalase, and mitochondrial hemoproteins that sustain detoxification, respiration, and redox buffering [38,43]. Another major source of heme processed in the liver arises from aged and damaged erythrocytes, which are continuously cleared from circulation [49,50,51,52]. Hemoglobin is synthesized in erythroid precursor cells in the bone marrow prior to erythrocyte maturation [11,48], providing the source of heme subsequently recycled in the liver.

In hepatic heme degradation, endothelial cells and Kupffer cells provide a complementary degradative arm of the system by clearing erythrocyte-derived heme through erythrophagocytosis, as well as heme released from circulating hemoproteins and basal cellular turnover, via heme oxygenase–dependent catabolism, thereby linking heme turnover to iron recycling [49,50,51,52]. This coupling between parenchymal synthesis and macrophage-mediated clearance supports continuous heme flux while minimizing accumulation of reactive heme and porphyrin species. Under physiological conditions, PPIX remains low, consistent with efficient flux completion. Kupffer cells thus represent a homeostatic macrophage population specialized for hepatic heme surveillance and recycling.

### 4.2. Inflammation: Resolution State

During inflammation, the spatial organization of hepatic heme metabolism is largely preserved but functionally reoriented toward enhanced degradation and turnover (Figure 3B). Liver-resident macrophages (Kupffer cells) upregulate HMOX1 in response to inflammatory and oxidative cues, increasing heme catabolism and coupling it to iron sequestration, antioxidant defense, and cytoprotective signaling [19,51,52,53]. This induction of the HMOX1 pathway represents a central adaptive response to heme-mediated stress [19,20].

Hepatocytes, in parallel, retain core metabolic functions, including mitochondrial respiration and CYP-mediated metabolism, although these processes may be quantitatively modulated under inflammatory conditions [38]. Importantly, heme biosynthesis is not fully suppressed, allowing continued flux through the pathway.

The net effect is a system characterized by elevated heme turnover, in which increased degradation capacity matches or exceeds production. This coordinated response limits the accumulation of free heme and reactive upstream porphyrin intermediates, thereby preserving redox homeostasis despite inflammatory stress. In this context, hepatocyte-derived heme flux remains functionally coupled to macrophage-mediated degradation [38], sustaining a dynamic intercellular metabolic loop.

We refer to this coordinated condition as a resolution state, in which inflammatory stress is managed through regulated turnover and clearance rather than intermediate accumulation. In this state, enhanced degradation acts not as a failure of metabolism, but as an active mechanism of metabolic stabilization.

### 4.3. Cancer: Expansion State

In HCC, the coordinated system of hepatic heme metabolism becomes progressively disrupted [33]. Transformed hepatocytes exhibit impaired flux completion, frequently associated with reduced FECH activity and consequent accumulation of PPIX [14,17,25]. This phenotype is supported by both biochemical studies and clinical observations using ALA–based imaging [13,14,15], in which tumor tissues preferentially accumulate PPIX due to incomplete conversion to heme (Figure 3C).

Concurrently, liver macrophages undergo functional reprogramming into tumor-associated macrophages (TAMs), which display altered iron handling, redox regulation, and metabolic phenotypes [34,44,46,51]. While resident healthy Kupffer cells are specialized for coordinated heme degradation and iron recycling under homeostatic conditions, TAMs represent a distinct activation state in which these processes are modified, contributing to altered coordination between heme production and clearance within the tumor microenvironment.

Within this context, the balance between hepatocyte-derived heme synthesis and macrophage-mediated degradation is likely perturbed. Tumor-associated changes in both hepatocyte metabolism and macrophage function may reduce effective degradative capacity relative to ongoing pathway activity, resulting in a functional uncoupling of synthesis and clearance. At the same time, tumor cells upregulate heme and porphyrin transport systems, including the heme exporter feline leukemia virus subgroup C receptor 1 FLVCR1 [22,24,54,55], facilitating redistribution of heme and intermediate metabolites into the surrounding microenvironment.

The resulting metabolic state is characterized by accumulation and spatial heterogeneity of porphyrin intermediates, together with redistribution of heme-related metabolites beyond the cell of origin. We define this condition as an expansion state, in which incomplete flux resolution, combined with altered intercellular coordination, permits intermediate pools to persist, diversify, and propagate within the tissue.

## 5. Unified Model: States of Heme Metabolism

The observations across pathway dynamics, functional utilization, and spatial organization converge on a unified model in which hepatic heme metabolism operates across distinct regimes defined by the fate of metabolic flux and the behavior of intermediates. This framework does not introduce new molecular components; rather, it reinterprets established biology through a unifying principle: whether metabolic flux is efficiently resolved into functional heme or remains incomplete, leading to upstream accumulation of reactive intermediates.

In the resolution state, most clearly observed during inflammation, metabolic flux is either efficiently completed or actively drained. Heme production in hepatocytes remains functionally balanced and integrated with downstream utilization. In parallel, macrophage-mediated degradation, driven by HMOX1, supports continuous turnover and removal of excess heme [19,49,51]. This coordination between production and intercellular compartmental activity limits the accumulation of upstream porphyrins, such that PPIX remains low even under increased metabolic demand. As a result, redox balance is preserved, and the system maintains stability through effective coupling between synthesis, utilization, and degradation, enabling it to absorb perturbations without loss of control.

In contrast, the expansion state, exemplified in cancer, arises when flux completion becomes impaired. Disruption at the terminal step of heme biosynthesis, particularly reduced FECH activity, combined with relative preservation or enhancement of upstream pathway steps, leads to accumulation of intermediates, most notably PPIX, reflecting a bottlenecked pathway [24,25,56]. This accumulation is often spatially heterogeneous [23], likely shaped by local variation in mitochondrial function, enzyme activity, and substrate availability. At the same time, clearance mechanisms may become insufficient or functionally uncoupled from synthesis, allowing intermediate pools to persist and expand. The resulting system is characterized by reduced or selectively reallocated functional heme output, while upstream pathway activity remains ongoing, contributing to sustained redox stress and metabolic instability.

Importantly, these regimes represent conceptual extremes along a continuum rather than strictly discrete states. They are defined not by absolute levels of heme, but by the relationship between metabolic flux and its completion. In the resolution state, intermediates remain transient and tightly controlled; in the expansion state, they persist and accumulate. Thus, the distinction between the “queen” and the “dark twin” reflects not different pathways, but alternative outcomes of the same pathway operating under different constraints. Heme metabolism is therefore defined not only by its end products, but also by the dynamic behavior of its intermediates.

## 6. Disease Interpretation Through the Intermediate Lens

Viewing heme metabolism through the behavior of intermediates reframes liver disease as a problem of flux resolution rather than simple pathway presence or absence [8,18,57]. Across diverse conditions, the same biosynthetic machinery is engaged, yet outcomes diverge depending on where flux is constrained and whether intermediates are efficiently cleared or permitted to accumulate. This perspective places PPIX and related porphyrin intermediates at the center of disease interpretation, as functional indicators of incomplete metabolic resolution.

Porphyrias represent the most direct manifestation of this principle. The hepatic porphyrias, including acute intermittent porphyria, variegate porphyria, and hereditary coproporphyria, arise from inherited defects in specific enzymes of the heme biosynthetic pathway [58,59,60]. These genetic disruptions impair flux at defined enzymatic steps, leading to the accumulation of upstream intermediates, including PPIX and related porphyrins, which are responsible for the characteristic clinical features. In this context, expansion of the intermediate pool is not secondary but intrinsic to the pathway, providing a canonical example in which metabolic flux fails at discrete enzymatic steps and disease emerges directly from intermediate accumulation.

More common liver diseases can be interpreted as partial or context-dependent manifestations of the same underlying principle. In metabolic liver disease, including nonalcoholic fatty liver disease (NAFLD) and nonalcoholic steatohepatitis (NASH), mitochondrial dysfunction and redox imbalance impair the efficiency of metabolic flux completion [61,62,63]. Although these conditions do not typically result in the accumulation of porphyrin intermediates observed in porphyrias, they exhibit features consistent with a partial expansion state, in which pathway coordination is weakened and heme homeostasis, particularly the coupling between hepatocytes and stromal compartments, becomes less tightly regulated [34,64,65]. In this setting, metabolic flux is not fully blocked but is inefficiently and variably resolved, contributing to oxidative stress and metabolic heterogeneity.

Drug-induced liver injury (DILI) represents a distinct mode of metabolic disruption in which demand for heme-dependent detoxification exceeds biosynthetic and regulatory capacity [5,66,67]. Induction of CYP enzymes increases heme utilization, placing additional demand on the heme biosynthetic pathway and creating a mismatch between pathway input and functional output. Under these conditions, metabolic flux becomes strained, leading primarily to depletion of functional heme pools and impaired coordination across the pathway.

In certain contexts, particularly when drug exposure both increases heme demand and perturbs specific enzymatic steps, accumulation of upstream intermediates may occur, as exemplified by drug-triggered porphyrias [68,69]. However, such accumulation is not a consistent feature of DILI, which more commonly reflects a demand-driven imbalance rather than sustained intermediate expansion. This imbalance contributes to oxidative stress, impaired detoxification capacity, and hepatocellular injury.

HCC represents a more pronounced expansion state. In this setting, impaired flux completion is associated with the accumulation of PPIX, reprogramming of heme utilization, and spatial heterogeneity of metabolic activity [8,57,70,71,72]. Despite this impairment, the pathway remains active but incompletely resolved, generating a persistent pool of labile heme and upstream intermediates that both reflect and contribute to tumor physiology. This state is further characterized by selective allocation of a constrained heme pool and increased metabolic heterogeneity within the tumor microenvironment [22,24,25].

Across these conditions, a common principle emerges: disease is not defined by whether the heme pathway is present or absent, but by where and how flux fails.

## 7. Translational Implications: Reading and Targeting the State

If metabolic state is encoded in the behavior of pathway intermediates, it becomes both measurable and potentially targetable. The accumulation of PPIX provides a functional readout of incomplete flux resolution and defines the expansion state in a manner that is both spatially and functionally informative. This principle underlies established clinical approaches such as ALA–induced fluorescence imaging, in which exogenous pathway input reveals tumor-selective PPIX accumulation and enables real-time visualization of malignant tissue [14].

More broadly, quantification of circulating or tissue-derived intermediates may offer a complementary strategy to assess metabolic state beyond static measurements of enzyme expression or end-product abundance. By capturing the dynamic behavior of metabolic flux, such approaches [73,74,75,76] have the potential to provide a more functionally relevant readout of pathway activity across physiological and disease contexts.

Therapeutically, this framework shifts emphasis from simple pathway inhibition toward restoration of flux resolution. Targeting the terminal step of heme biosynthesis, such as through modulation of FECH [11,12,77,78], may help relieve the bottleneck that characterizes the expansion state and reduce the accumulation of upstream intermediates. In parallel, enhancing heme degradation via the heme oxygenase-1 (HO-1) axis may promote a transition toward a resolution state by increasing heme turnover and supporting redox homeostasis [20,79,80]. Importantly, these strategies do not seek to uniformly increase or suppress pathway activity; rather, they aim to restore coordination between synthesis, utilization, and clearance, thereby reestablishing balanced metabolic flux.

The spatial dimension of this system introduces additional therapeutic opportunities. In cancer, increased expression of transporters such as FLVCR1 is consistent with active export of heme and porphyrin intermediates into the tumor microenvironment [54,55]. This suggests that intermediate accumulation is not confined to individual cells but may be redistributed across tissue compartments. In parallel, macrophages are reprogrammed into TAMs, altering their capacity for heme degradation and iron handling [34,44,46,51]. These changes collectively point to a loss of coordinated intercellular coupling in heme metabolism. Targeting these processes, either by modulating metabolite export or by restoring macrophage-mediated clearance, may provide a strategy to reestablish metabolic coordination across the tumor ecosystem.

Together, these strategies reflect a shift toward an intermediate-aware view of metabolism, in which diagnosis and therapy are guided not only by pathway components, but by the state of flux and its spatial organization.

## 8. Conclusions: The Queen Revealed by the Dark Twin

Heme metabolism in the liver is best understood not as a linear biosynthetic pathway, but as a state-dependent system in which completion and failure coexist within the same chemical network [18,25,32,57]. Heme represents completion, an integrated cofactor that supports respiration, detoxification, and redox control. In contrast, PPIX reflects incomplete resolution [17,24,25,74], emerging as a reactive intermediate when metabolic flux is not fully resolved. These two outcomes, the “queen” and the “dark twin”, are not distinct entities, but alternative states of the same pathway operating under different constraints.

This framework highlights three organizing principles. First, metabolic state is defined by flux: whether pathway activity is efficiently completed or stalls upstream. Second, it is shaped by space: hepatocyte-driven synthesis and utilization must be coordinated with macrophage-mediated degradation and recycling. Third, it depends on coupling: the balance between these compartments determines whether intermediates are cleared or allowed to accumulate. When flux is resolved and coupling is maintained, the system operates in a state of integration; when flux is incomplete and coupling is disrupted, intermediates expand, and metabolic instability emerges.

By focusing on the behavior of intermediates, this model links pathway dynamics, cellular function, and tissue organization into a unified view of liver metabolism. It provides a conceptual bridge between biochemical mechanisms and disease states, suggesting that metabolic dysfunction is best understood not by the absence of pathways, but by the conditions under which they fail to resolve.

The queen defines order, but it is the dark twin that reveals when the system begins to fail.

## Data Availability

All information has been included in the manuscript.

## Abbreviations

The following abbreviations are used in this manuscript:

5-ALA	5-Aminolevulinic acid
ALA	Aminolevulinic acid
ALAS1	Aminolevulinate synthase 1
CAT	Catalase
CYP	Cytochrome P450
CYC1	Cytochrome c1
DILI	Drug-induced liver injury
ETC	Electron transport chain
FECH	Ferrochelatase
FLVCR1	Feline leukemia virus subgroup C receptor 1
HCC	Hepatocellular carcinoma
HMBS	Hydroxymethylbilane synthase
HMOX1 (HO-1)	Heme oxygenase-1
NAFLD	Nonalcoholic fatty liver disease
NASH	Nonalcoholic steatohepatitis
PDT	Photodynamic therapy
PPIX	Protoporphyrin IX
TAMs	Tumor-associated macrophages
